# Improved Preprocedure Hematocrit in Chronic RBC Exchange Patients Following Splenectomy: A Case Report

**DOI:** 10.1155/crh/9580128

**Published:** 2025-10-08

**Authors:** Aswath P. Chandrasekar, Scott A. Koepsell, Shelly M. Williams, Aleh Bobr

**Affiliations:** ^1^Department of Pathology, Microbiology and Immunology, University of Nebraska Medical Center, Omaha, Nebraska, USA; ^2^Department of Laboratory Medicine and Pathology, Mayo Clinic, Rochester, Minnesota, USA

**Keywords:** apheresis, beta thalassemia, isovolemic hemodilution, red cell exchange, sickle cell disease, splenectomy, transfusion refractoriness

## Abstract

**Background:**

Chronic RBC exchange (RCE) is an established therapeutic strategy used to prevent the development of serious complications in patients with sickle cell disease and beta thalassemia. A subset of these patients have an accelerated decline of transfused red cells, leading to suboptimal exchange transfusions since the preprocedure hematocrit (HCT) is too low to allow for isovolemic hemodilution. These patients often have concomitant splenomegaly.

**Methods:**

In our institution, we had 3 patients who had rapid decline in HCT post-RCE and who underwent a splenectomy. We compared the pre- and postsplenectomy hemoglobin S and HCT values for two patients with sickle cell anemia and one with beta thalassemia, undergoing chronic RCE.

**Results:**

We observed a significant increase in the preprocedure HCT, from a mean ± SD of 21.11 (±2.5) presplenectomy to 25.02 (±1.8) postsplenectomy (*p* < 0.0001). This was accompanied by a significant increase in the interval number of days between procedures, from 29.6 (±5.6) days to 34.8 (±7.2) days following splenectomy (*p*=0.0046). Comparing pre- and postsplenectomy HCT values to the threshold HCT value required for isovolemic hemodilution (HCT = 23%) revealed that splenectomy resulted in a highly significant increase (*p* < 0.0001) above the threshold.

**Discussion:**

Our observations here suggest that in a subset of patients, splenomegaly may result in accelerated decline of transfused red cells which improves following splenectomy, resulting in improved clinical parameters and more efficient RCE.

## 1. Introduction

Sickle cell anemia and β-thalassemia are inherited hemoglobinopathies occurring as a result of genetic abnormalities in the β-globin gene. At present, stem cell transplantation remains the only known curative therapy, one which is limited in its scope and applicability to most patients. Sickle cell anemia occurs as a result of an adenine to thymine substitution in the HBB gene, leading to the replacement of glutamic acid with valine at Position 6 of the beta-globin protein, resulting in the production of a variant of hemoglobin—hemoglobin S (HbS) [1]. In the deoxygenated state, HbS undergoes polymerization [2], leading to the aberrant “sickle” red blood cell (RBC) morphology. The clinical manifestations of sickle cell anemia are a result of the increased propensity of the sickled RBCs to aggregate with platelets and neutrophils leading to vaso-occlusion and end-organ damage [1, 3]. Within this paradigm, studies have now clearly established the beneficial role of chronic red cell transfusion in the prevention of stroke, the normalization of clinical parameters and amelioration of vaso-occlusive crises [4, 5]. It has also been illustrated that the cessation of transfusion results in a return to baseline for these patients [6]. Similarly β-thalassemia, which occurs due to impaired β-globin synthesis, presents with chronic microcytic anemia with varying degrees of clinical severity [7]. Over three-hundred and fifty mutations have been described to result in β-thalassemia, with the most severe variants resulting in a complete absence of β-globin synthesis. The absence of β-globin chain incorporation into hemoglobin results in the formation of unstable α-globin tetramers which lead to hemolysis. Severe hemolysis and ineffective erythropoiesis necessitate chronic transfusions in a subset of patients [7]. Splenomegaly is encountered in both disorders and has been demonstrated to result in the need for increased transfusion support and chronic transfusions [8, 9].

The widespread adoption of the isovolemic hemodilution red cell exchange (RCE) technique has now allowed clinicians to address two of the most common complications of chronic red cell transfusions—iron overload and antigen sensitization—by reducing the overall number of units transfused per procedure [10]. The major determinant of the utility of isovolemic hemodilution is the preprocedure hematocrit (HCT). A subset of patients who receive chronic RCEs demonstrate an accelerated decline of transfused red cells, characterized by a low preprocedure HCT, leading to suboptimal exchange transfusions. Herein, we describe two patients with sickle cell anemia and one patient with β-thalassemia, with splenomegaly, who demonstrated accelerated decline of transfused RBCs in the absence of red cell antibodies, who underwent therapeutic splenectomy with improvement in their hematological parameters.

## 2. Methods

The data presented here are anonymized and represent retrospective review of laboratory tests. According to IRB rules, an IRB review is not required in this situation. The data presented here do not constitute a clinical trial. No written consent has been obtained from the patients as there are no patient identifiable data included in this case report/series.

We reviewed the preprocedure HCT and HbS levels for three patients who were receiving chronic RCE at our institution between September 2019 and November 2022, who had undergone splenectomy. Analysis of hemoglobin was performed using a combination of liquid chromatography (Trinity Ultra 2) and capillary electrophoresis (Sebia CAPILLARYS 2) due institutional change of method. RBC antibodies were ruled out for these patients. The pre- and postsplenectomy HbS and HCT values were compared using one-sample *T*-tests, two-tailed, unpaired, *T*-tests, or Mann–Whitney tests, as appropriate. GraphPad PRISM 10.0 was used to plot the data and calculate statistical significance, and a *p* value less than 0.05 was considered significant.

Apheresis procedures: All exchange procedures were performed using the Spectra Optia apheresis system, Version 12.0 (Terumo Blood And Cell Technologies, Lakewood, CO, USA). The anticoagulant used was anticoagulant citrate dextrose solution (ACD-A). The interval between RCE in our institution is determined by the longest time possible to maintain HbS below 50% (patient specific and varies between 4 and 8 weeks) for sickle cell patients and ability to maintain 9.5 g/dL hemoglobin preprocedure for thalassemia patients. The fraction of cells remaining (FCR) for RCE is set 30% for all procedures. RBC units for RCE are Rh and Kell matched for the patients without alloantibody (all the patients described here), with an assumed HCT of the units of 60%. RBC units for RCE are 14 days or younger.

## 3. Results

**Figure 1:**
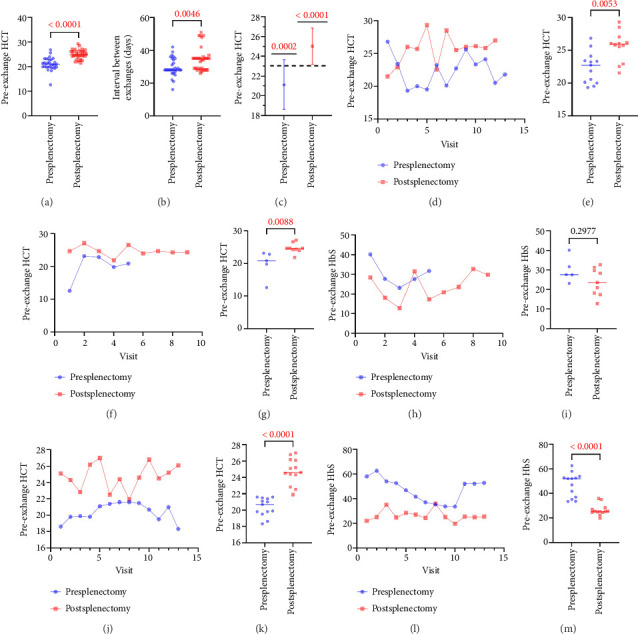
Splenectomy results in improved preprocedure hematological parameters in patients receiving chronic red cell exchange. We reviewed the preprocedure hematocrit and hemoglobin S percentages for three patients who were receiving chronic red cell exchange at our institution between September 2019 and November 2022. (a) Pooled data of the pre-RCE hematocrit before and after undergoing splenectomy. (b) Mean interval between RCE procedures (*n* = 3). (c) Splenectomy resulted in a highly significant increase (*p* < 0.0001) in hematocrit values, relative to the 23% threshold for hemodilution, significance calculated using a one sample *T*-test. (d, e) Patient 1, diagnosed with β-thalassemia, demonstrated significant improvement in pre-RCE hematocrit following splenectomy. (f, g) Pre-RCE hematocrit for Patient 2, diagnosed with sickle cell anemia, demonstrate significant improvement following splenectomy. (h, i) Pre-RCE hemoglobin S percentage before and after splenectomy, showing a nonsignificant decrease. (j, k) Pre-RCE hematocrit for Patient 3, diagnosed with sickle cell anemia, demonstrate significant improvement following splenectomy, accompanied by (l, m) significant reduction in pre-RCE hemoglobin S percentage. Statistical significance was calculated using unpaired, *T*-tests, or Mann–Whitney tests, as appropriate. *p* < 0.05 was considered significant.

Across all three of our patients, we observed a significant increase in the preprocedure HCT, from a mean ± SD of 21.11 (±2.5) presplenectomy to 25.02 (±1.8) postsplenectomy (Figure 1(a); *p* < 0.0001). This was accompanied by a significant increase in the interval number of days between procedures, from 29.6 (±5.6) days to 34.8 (±7.2) days following splenectomy (Figure 1(b); *p*=0.0046). Given that a preprocedure HCT value of 23% is the threshold value utilized to determine whether isovolemic hemodilution is possible [10], we aimed to see if splenectomy significantly affected the HCT value relative to this threshold. We utilized a one-sample *T* test, comparing the pre- and postsplenectomy HCT values to the hypothetical value of 23 and observed that splenectomy resulted in a highly significant increase (Figure 1(c); *p* < 0.0001) of HCT values, relative to this threshold.

We subsequently examined the effect of splenectomy for each patient individually.

Patient 1 (male, 12 years old at the time of splenectomy), diagnosed with β-thalassemia major (C316-197T mutation and C126 deletion), received 13 exchange transfusions prior to- and 12 exchange transfusions following splenectomy at the time of this study (Figure 1(d)) and demonstrated significant improvement in preprocedure HCT (Figure 1(e); Table 1). Splenectomy indication was splenomegaly, increased chronic hemolysis, and poor transfused RBC survival.

Patient 2 (male, 8 years old at the time of splenectomy), diagnosed with sickle cell anemia (SS-disease), received 5 exchange transfusions prior to and 9 exchange transfusions following splenectomy at the time of this study (Figure 1(f)) and demonstrated significant improvement in preprocedure HCT (Figure 1(g); Table 1). One RBC exchange procedure was omitted from the analysis as it was performed in the setting of splenic sequestration crisis, concurrent transfusions, and acute clinical setting. There was no significant difference in the level of the preprocedure HbS since interprocedure interval was adjusted postsplenectomy by adding additional week, thus resulting in the HbS rising to comparable presplenectomy levels, while still maintaining higher HCT to allow for isovolemic hemodilution (Figures 1(h) and 1(1); *p*=0.2977). Splenectomy indication was a splenic sequestration crisis.

Patient 3 (male, 17 years old at the time of splenectomy), also diagnosed with sickle cell anemia (SS-disease), received 13 exchange transfusions prior to and 13 exchange transfusions following splenectomy at the time of this study (Figure 1(j)) and demonstrated significant improvement in preprocedure HCT (Figure 1(k); Table 1). This was accompanied by a significant decrease in preprocedure HbS percentage, from 47.05 (±9.8) to 26.39 (±4.5) following splenectomy (Figure 1(l) and 1(m); *p* < 0.0001). Splenectomy indication was splenomegaly, increased chronic hemolysis, poor transfused RBC survival. A summary of the pre- and postsplenectomy values for each patient is provided in Table 1.

## 4. Discussion

Overall, our findings described here suggest that, in patients receiving chronic RCE for transfusion-dependent β-thalassemia and sickle cell anemia, concomitant splenomegaly may be resulting in more pronounced decline of transfused red cells, which improve following therapeutic splenectomy.

The spleen contributes to the clearance of transfused RBCs, in part, through macrophage-mediated RBC phagocytosis [11, 12]. Splenectomy has historically been a relevant therapeutic avenue for patients with sickle cell anemia in the context of splenic abscess, sequestration, hypersplenism and massive splenomegaly [13]; however, there is limited evidence suggesting an overall survival benefit [14]. Similarly, in β-thalassemia, hypersplenism and massive splenomegaly are indications for splenectomy, along with increased annual blood requirement more than 1.5 times that of splenectomized patients [15]. Clinical assessment is required in determining the utility of splenectomy as a therapeutic modality for either sickle cell or β-thalassemia, due to the increased susceptibility to infections [16], venous thromboembolism [17, 18], and risk of surgical complications.

Our observations here suggest that in addition to providing clinical benefit—in the form of improvement in anemia as a result of increased RBC survival, decrease in chronic hemolysis, and level of bilirubin—therapeutic splenectomy may also inadvertently benefit the patient through the improvement of RCE efficiency, through better spacing of procedures (5 vs. 4 weeks) and ability to perform IHD. Across our small cohort, we observed a significant increase in preprocedure HCT to a mean value above the 23% threshold, which would allow the pursual of isovolemic hemodilution for these patients. In our patients with sickle cell anemia, we observed a lower mean HbS percentage in both patients, following splenectomy, though this was only statistically significant for Patient 3.

Our study is limited by its small sample size, which prevents definitive correlations between splenectomy and clinical prognosis. Furthermore, as splenectomy can be accompanied by several severe complications as detailed above, the applicability of splenectomy as a therapeutic strategy may be limited to a few patients. Future, long-term observations are needed to assess whether the benefits of decreased transfusion requirements and iron overload outweigh the risks of splenectomy.

In summary, our findings in this study suggest that chronically transfused patients with concomitant splenomegaly and accelerated decline in transfused RBCs demonstrate improved retention of transfused red cells and improved RCE efficiency following splenectomy.

## Figures and Tables

**Table 1:** A summary of the pre- and postsplenectomy hematocrit (%) and hemoglobin S (%).

	Hematocrit (%)		Hemoglobin S (%)	
	Presplenectomy (mean ± SD)	Postsplenectomy (mean ± SD)	Presplenectomy (mean ± SD)	Postsplenectomy (mean ± SD)
Patient 1	22.33 (±2.4)	25.57 (±2.3)	N/A	N/A
Patient 2	19.88 (±4.3)	24.71 (±1.5)	30.02 (±6.5)	23.84 (±7.1)
Patient 3	20.37 (±1.1)	24.72 (±1.6)	47.05 (±9.8)	26.39 (±4.5)
Pooled data	21.11 (±2.5)	25.02 (±1.8)	42.32 (±11.8)	25.35 (±5.7)

*Note:* Data expressed as (Mean ± SD).

## References

[1] Kato G. J., Piel F. B., Reid C. D. (2018). Sickle Cell Disease. *Nature Reviews Disease Primers*.

[2] Kaperonis A. A., Handley D. A., Chien S. (1986). Fibers, Crystals, and Other Forms of HbS Polymers in Deoxygenated Sickle Erythrocytes. *American Journal of Hematology*.

[3] Ware R. E., de Montalembert M., Tshilolo L., Abboud M. R. (2017). Sickle Cell Disease. *The Lancet*.

[4] Lee M. T., Piomelli S., Granger S. (2006). Stroke Prevention Trial in Sickle Cell Anemia (STOP): Extended Follow-Up and Final Results. *Blood*.

[5] Adams R. J., McKie V. C., Hsu L. (1998). Prevention of a First Stroke by Transfusions in Children with Sickle Cell Anemia and Abnormal Results on Transcranial Doppler Ultrasonography. *New England Journal of Medicine*.

[6] Adams R. J., Brambilla D. (2005). Discontinuing Prophylactic Transfusions Used to Prevent Stroke in Sickle Cell Disease. *New England Journal of Medicine*.

[7] Taher A. T., Musallam K. M., Cappellini M. D. (2021). β-Thalassemias. *New England Journal of Medicine*.

[8] Rivella S. (2015). β-Thalassemias: Paradigmatic Diseases for Scientific Discoveries and Development of Innovative Therapies. *Haematologica*.

[9] Nardo‐Marino A., Glenthøj A., Brewin J. N. (2022). The Significance of Spleen Size in Children With Sickle Cell Anemia. *American Journal of Hematology*.

[10] Matevosyan K., Anderson C., Sarode R. (2012). Isovolemic Hemodilution-Red Cell Exchange for Prevention of Cerebrovascular Accident in Sickle Cell Anemia: the Standard Operating Procedure. *Journal of Clinical Apheresis*.

[11] Roussel C., Buffet P. A., Amireault P. (2018). Measuring Post-Transfusion Recovery and Survival of Red Blood Cells: Strengths and Weaknesses of Chromium-51 Labeling and Alternative Methods. *Frontiers of Medicine*.

[12] Sosale N. G., Rouhiparkouhi T., Bradshaw A. M., Dimova R., Lipowsky R., Discher D. E. (2015). Cell Rigidity and Shape Override CD47’s “self”-signaling in pPagocytosis by hHperactivating myosin-II. *Blood*.

[13] Katz S. C., Pachter H. L. (2006). *Indications for Splenectomy*.

[14] Owusu-Ofori S., Remmington T. (2017). Splenectomy Versus Conservative Management for Acute Sequestration Crises in People with Sickle Cell Disease. *Cochrane Database of Systematic Reviews*.

[15] Cappellini M. D., Cohen A., Eleftheriou A., Piga A., Porter J., Taher A. (2008). *Guidelines for the Clinical Management of Thalassaemia*.

[16] Eraklis A. J., Kevy S. V., Diamond L. K., Gross R. E. (1967). Hazard of Overwhelming Infection After Splenectomy in Childhood. *New England Journal of Medicine*.

[17] Crary S. E., Buchanan G. R. (2009). Vascular Complications After Splenectomy for Hematologic Disorders. *Blood*.

[18] Cappellini M. D., Robbiolo L., Bottasso B. M., Coppola R., Fiorelli G., Mannucci A. P. (2000). Venous Thromboembolism and Hypercoagulability in Splenectomized Patients with Thalassaemia Intermedia. *British Journal of Haematology*.

